# Mechanotransduction Regulates the Interplays Between Alveolar Epithelial and Vascular Endothelial Cells in Lung

**DOI:** 10.3389/fphys.2022.818394

**Published:** 2022-02-18

**Authors:** Chuyang Lin, Xiaolan Zheng, Sha Lin, Yue Zhang, Jinlin Wu, Yifei Li

**Affiliations:** Key Laboratory of Birth Defects and Related Diseases of Women and Children of MOE, Department of Pediatrics, West China Second University Hospital, Sichuan University, Chengdu, China

**Keywords:** mechanotransduction, alveolar epithelial cells, vascular endothelial cells, pulmonary diseases, interplays

## Abstract

Mechanical stress plays a critical role among development, functional maturation, and pathogenesis of pulmonary tissues, especially for the alveolar epithelial cells and vascular endothelial cells located in the microenvironment established with vascular network and bronchial-alveolar network. Alveolar epithelial cells are mainly loaded by cyclic strain and air pressure tension. While vascular endothelial cells are exposed to shear stress and cyclic strain. Currently, the emerging evidences demonstrated that non-physiological mechanical forces would lead to several pulmonary diseases, including pulmonary hypertension, fibrosis, and ventilation induced lung injury. Furthermore, a series of intracellular signaling had been identified to be involved in mechanotransduction and participated in regulating the physiological homeostasis and pathophysiological process. Besides, the communications between alveolar epithelium and vascular endothelium under non-physiological stress contribute to the remodeling of the pulmonary micro-environment in collaboration, including hypoxia induced injuries, endothelial permeability impairment, extracellular matrix stiffness elevation, metabolic alternation, and inflammation activation. In this review, we aim to summarize the current understandings of mechanotransduction on the relation between mechanical forces acting on the lung and biological response in mechanical overloading related diseases. We also would like to emphasize the interplays between alveolar epithelium and vascular endothelium, providing new insights into pulmonary diseases pathogenesis, and potential targets for therapy.

## Introduction

As an area of gas exchange, the lung has extensive vascular and bronchial–alveolar networks and displays tension properties after birth. Accordingly, mechanical stimulation contributes significantly to maintaining the normal development of pulmonary tissue as well as its functional homeostasis. The adaptation to mechanical stress is differentially regulated among various parts of the human body, with surrounding microenvironments providing different physical stimuli that activate sensory transduction signaling pathways in a tissue-specific manner. The transformation of external mechanical forces into intracellular signaling is called mechanotransduction. There are two common types of mechanosensors—biophysical and biochemical—located on the cell membrane. Biophysical sensors help connect the extracellular matrix (ECM) with the actin cytoskeleton and reshape actin proteins and the nuclear membrane, ultimately leading to altered chromosomal structure and patterns of gene expression. Meanwhile, chemical sensors primarily influence the modification of downstream molecules, converting external biophysical signals into intracellular biochemical ones. A stable mechanical environment is required for development, especially during tissue specialization. Notably, the pulmonary microenvironment is consisted with airway structure and vascular vessels. The alveolar epithelium and vascular endothelium are supposed to subject different injuries under various pathological mechanical conditions. However, the stimulation from either epithelial or endothelial cells would remodel the gas exchange environment in consequence and affects each other *via* their communications. Thus, there are several interplays between alveolar epithelial and endothelial cells in mechanotransduction manner. Moreover, mesenchymal transition could both be identified in epithelial (EMT) and endothelial (EndMT) cells. So that, we would like to summarize the physiobiological changes under pathological mechanical stress for lung epithelial and endothelial cells, and also try to demonstrate how they influence each other.

## Mechanical Stress is Critical During Pulmonary Development and Functional Homeostasis

The lung is composed of epithelial cells such as the type I and type II alveolar cells (AT1 and AT2 cells), endothelial cells of the arteries, veins, and capillaries, stromal cells, and multiple immune cell types such as the monocytes and macrophages (Travaglini et al., 2020). Different cell types in the lungs vary in their ability to withstand mechanical forces during respiration and pulmonary blood flow. The alveolar unit is the basic functional unit in the lung for gas exchange and is mostly composed of AT1, AT2, and capillary endothelial cells, as well as monocytes and macrophages (Spieth et al., 2014).

During early pregnancy, the pulmonary luminal volume in the fetus is significantly low. The pulmonary pressure gradient of the lung fluid increases the elasticity of the fetal lung tissues and stimulates the lung epithelial cells to actively secrete chloride ions. These chloride ions are transported into the stroma and the lung cavity by blood. The chloride ions move along a concentration gradient toward the lumen through the chloride channels to form the lung fluid, which prevents the amniotic fluid from entering the airways; the lung fluid also removes mucus and other cell debris from the airway cavity (Cotten, 2017). In the animal models, lung fluid secretion increases when the pressure in the lumen drops below that of the amniotic fluid. Reduced amniotic fluid volume increases the gradient between intraluminal pressure and amniotic fluid pressure. This increases the lung fluid outflow and reduces the expansion pressure in the lumen and the concentrations of various growth and maturation factors (Najrana et al., 2017, 2021). The growth factors are released when the lung tissues stretch in response to the fetal breathing movements, and stimulate the proliferation and differentiation of the epithelial cells and the production of surfactants. Early mammalian airways exhibit spontaneous transient airway obstructions due to airway peristalsis. Peristaltic contractions and airway occlusions induce rhythmic stretching and relaxation of the growing buds by directing the fluid waves to the apex of the lung. Therefore, the airway peristalsis and obstruction generate pressure and extension of the developing lung tip (Jesudason, 2009).

Alveolar development begins before birth and continues until adolescence. Postnatal periodic stretching is a key determinant of lung size and is essential for septum extension. During the respiratory cycle, the lung matrix experiences cyclic stretching because of the periodic tension that is applied on the developing lung tissue. Based on *in vitro* experimental results, 5–12% cyclic stretching is considered as physiological stress, whereas ≥ 20% cyclic stretching is considered as pathological stress (Young et al., 2015). The synthesis and remodeling of the lung matrix is required for primary and compensatory lung growth. Lung elastase activity is dependent on the tensile strength. In the mice, elastase activity increases by two-fold during the alveolar stage of postnatal lung morphogenesis (Young et al., 2015). This demonstrates the effects of postnatal respiratory movement on alveolar development. Furthermore, periodic alveolar tension is mediated by the release of growth factors *via* multiple intracellular signal transduction pathways.

Endothelial cells, fibroblasts, and smooth muscle cells are continuously stimulated in the pulmonary arteries by mechanical forces such as shear stress and pulsatile blood pressure. Both shear stress and pulsatile blood pressure are altered under conditions of pulmonary hypertension (PH). In response to blood pressure, pulmonary arterial endothelial cells (PAECs) align longitudinally to form the inner tunica of the blood vessels, whereas the pulmonary arterial smooth muscle cells (PASMCs) align circumferentially to form the median layer. The composition of the ECM also contributes to arterial stiffness and modulates the mechanical forces acting on the vessel wall. ECM is secreted by the PASMCs and pulmonary artery adventitial fibroblasts (PAAF). ECM interacts with the cells through the stretch-activated channels (SAC) and receptors such as the integrins. Therefore, ECM plays a key role in the stiffness-dependent activation of vascular endothelial cells.

In general, the fetal lung is subjected to gradient lung fluid pressure and airway peristalsis. After birth, cyclic stretching stimulates lung development as a result of breathing movements and shear stress induced by blood flow, which act on the PAECs and the alveolar epithelium.

## Mechanotransduction in Aveolar Epithelial Cells and Vascular Endothelial Cells

Physical strain is characterized by the relative change in length in response to applied force. Alveolar epithelial cells are subjected to mechanical strain during breathing, whereas vascular endothelial cells are primarily affected by shear stress, strain, and hydrostatic pressure. Distinct types of cells in the lungs experience diverse mechanical forces based on their location. For example, the apical surface of the epithelium experiences shear stress from the fluid layer in the airways and the alveoli, whereas the basolateral surface of the epithelium experiences stretch or strain due to expansion of the basement membrane. Therefore, two different types of physical forces act on the same cell type (alveolar epithelial cells) and regulate distinct biological functions *via* signal transduction pathways (Garcia et al., 2006). Furthermore, contraction of the actin cytoskeleton induces tension that is transmitted throughout the cell including the nucleus. The pulmonary cells also interact with the surrounding environment through adhesion receptors such as the integrins, which function as a link between the cytoskeleton and the ECM. The cytoskeleton is an interconnected biopolymer network within the plasma membrane that exerts a centripetal force on the surrounding matrix.

**FIGURE 1:**
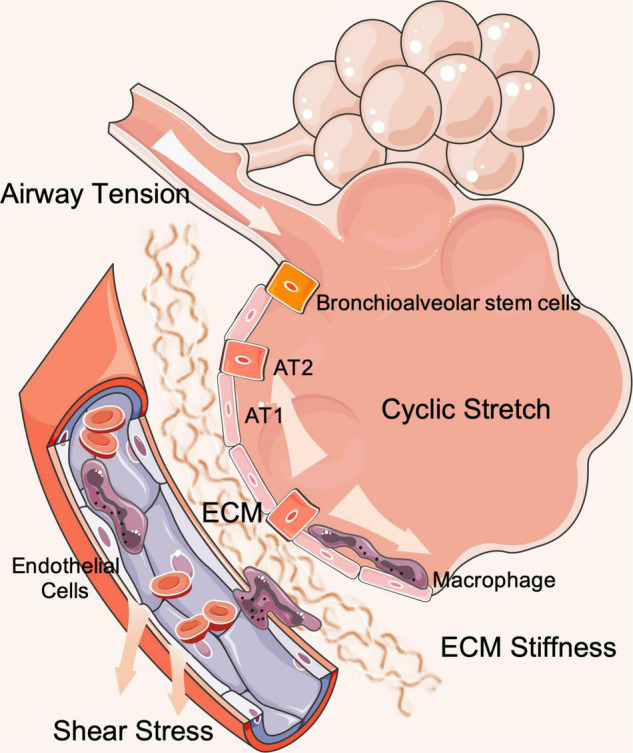
The schematic diagram of mechanical stresses loading on pulmonary alveoli and blood vessels. AT1, alveolar type 1 cells; AT2, alveolar type 2 cells; ECM, extracellular matrix.

Several studies have investigated the mechanisms that regulate cellular structure, function, proliferation, differentiation, secretion, movement, and metabolism in response to mechanical stimulation by physical forces (Liao et al., 2020; Liu et al., 2021). Aberrant mechanical stretching can result in cellular barrier dysfunction, metabolic dysfunction, cytotoxicity, and inflammation (Figure 1).

### Mechanical Forces Regulate the Homeostasis of Alveolar Cells

AT1 and AT2 are two different types of alveolar epithelial cells. AT1 cells do not show any proliferative capacity and are mainly involved in gas exchange, whereas, the AT2 cells can differentiate into AT1 cells and are the major source of pulmonary surface-active material. AT1 cells account for more than 95% of the alveolar surface area and respond to periodic stretches through genomic changes to modulate paracellular permeability.

Mechanical stretching promotes proliferation (Han et al., 2005), secretion and metabolism of surface-active substances, cellular damage or death (Arold et al., 2009), and migration (Desai et al., 2008) of AT2 cells. The proliferation of alveolar epithelial cells is essential for maintaining the integrity of the epithelium, especially during the process of repair after lung injury. Mechanical forces induce mitotic activity and growth factor synthesis and secretion by the alveolar epithelial cells (Noskovičová et al., 2015). AT2 cells subjected to periodic mechanical stress in FlexCell units display increased DNA synthesis after exposure to conditioned medium from lung fibroblasts compared to those cultured under static conditions. Mechanical strain also activates the expression of platelet-derived growth factor receptor beta (PDGFRB) in the lungs during development. Synthesis and secretion of pulmonary surfactants is the major biological function of the AT2 cells. Cyclic stretch stimulates the release of surfactant phospholipids from the AT2 cells by rapidly increasing the intracellular calcium ion concentrations.

**FIGURE 2:**
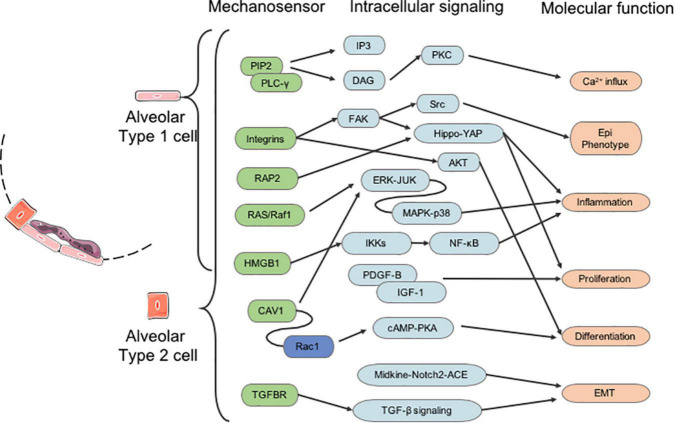
The molecular signaling of mechanotransduction in alveolar type 1 and type 2 (AT1 and AT2) cells. EMT, epithelial-mesenchymal transition.

Integrins, growth factor receptors, G-protein-coupled receptors, mechanoresponsive ion channels (e.g., Ca^2+^), and cytoskeletal strain responses are the main mediators of signal transduction pathways in response to changes in the extrinsic biochemical environment (Duscher et al., 2014). Furthermore, mechanical forces also transduce signals through several mechanosensitive focal adhesion proteins. The integrins transmit extracellular signals and induce intracellular cytoskeletal modifications (Figure 2).

#### Ion Channels

During fetal development, physiological stretching drives lung growth and maturation. The α-subunit of the alveolar epithelial sodium channel (ENaC) facilitates the clearance of lung fluid during the perinatal period. Mustafa et al. (2014) demonstrated that mechanical stretching induced the expression of ENaC *via* p38-mitogen-activated protein kinase (MAPK) and c-Jun N-terminal kinase (JNK).

Stretching also induces Ca^2+^ influx by activating the ion channels. TRPV4 and Piezo1 serve as the main ion channels that mediate the influx of Ca^2+^ ions in the alveolar epithelial cells. Liang et al. (2019) demonstrated that Piezo1 expression was significantly increased in the AT2 cells of patients with acute respiratory distress syndrome (ARDS); moreover, Piezo1 induced Bcl-2 dependent apoptosis *via* Ca^2+^ influx, but these effects were attenuated by inhibiting Piezo1. Mechanical stretching also induces the protein tyrosine kinases (PTK) to activate phospholipase C-γ (PLC-γ) through tyrosine phosphorylation (Liu et al., 2014). Active PLC-γ hydrolyzes phosphatidylinositol 4,5-bisphosphate (PIP2) into 1,4,5-trisphosphate (IP3) and diacylglycerol (DAG). IP3 mobilizes Ca^2+^ from the intracellular storage sites such as the endoplasmic reticulum (ER), whereas, DAG activates protein kinase C (PKC) in the presence of intracellular Ca^2+^ (Hsiao et al., 2016; Lu et al., 2021).

#### Integrins-FAK-MAPK-NF-κB

Activated integrins regulate several intracellular signaling molecules and pathways by recruiting focal adhesion proteins and the focal adhesion kinase (FAK). Besides, integrins function as a bridge between the F-actin cytoskeleton and the ECM and transduce signals generated through changes in mechanical stress. The integrin-Talin-F-actin-Nestin-SUN-nuclear lamina signaling axis modulates the nuclear envelope and induces transcriptional changes in response to the extracellular mechanical stress (Sun et al., 2016). Mechanical forces promote TRE-mediated gene expression *via* activation of RAS, ERK1/2, and JNK signaling pathways by reinforcing integrin-ECM binding (Parsons et al., 2010; Cho et al., 2017). Furthermore, p38-MAPK and several transcription factors downstream of various intrinsic signaling pathways are also activated, subsequently leading to the activation of NF-κB (Liu et al., 2016). Integrin family members also activate IκB kinases (IKKs), which induce the release of NF-κB from the cytoplasm to the nucleus in response to signals for maintaining lung development and alveolarization. MAPKs and NF-κB promote the transcription of several early response genes, which subsequently induce the transcription of inflammation-related genes *via* the cyclooxygenase (COX)-2/prostaglandin E2/Interleukin (IL)-8 signaling pathway (Dong et al., 2015).

#### Rho GTPase-YAP/TAZ

Rho-associated protein kinase (ROCK), myocardin-related transcription factor-A (MRTF-A), yes-associated protein 1 (YAP), and transcriptional coactivator with PDZ-binding motif (TAZ) are key players in the response of the alveolar epithelial cells to mechanical stimulation (Deng et al., 2020). Rho guanosine triphosphatase (GTPase) are a family of small G-proteins of the Ras superfamily such as Rac, Rho, and CDC42 are small GTP-binding signaling proteins that regulate cytoskeletal dynamics by mediating actin polymerization and myosin II-mediated contraction through FAK (Jiang et al., 2012; Maurer and Lammerding, 2019). RAP2a is a novel Rap GTPase that responds to mechanical stretch, but its actual function in the pulmonary system has not been established (Meng et al., 2018). Rho-mediated actin polymerization induces MRTF-A nuclear translocation, activation of α-smooth muscle actin (α-SMA) gene expression, and type I collagen synthesis (Ni et al., 2013).

#### Metabolic Status

Mechanical stress induces oxidative damage and ER stress in the alveolar epithelial cells, which release injury-related molecules with damage-associated molecular patterns (DAMPs) that trigger tissue repair and fibrotic response (Lionetti et al., 2005; Tanaka et al., 2017; Valentine et al., 2018). DAMPs and high mobility group box 1 (HMGB1) released by the injured tissues promote tissue repair and angiogenesis by inducing the migration and proliferation of stem cells (Yang et al., 2020). When regeneration is not successful, HMGB1 induces fibrosis by stimulating fibroblast activation and endothelial cell proliferation. Patel et al. (2020) demonstrated that hypoxia induced the activation of host macrophages *via* HMGB1, but these effects were attenuated by the dietary antioxidants.

### Mechanical Forces Regulate Functions of Vascular Endothelial Cells

**FIGURE 3:**
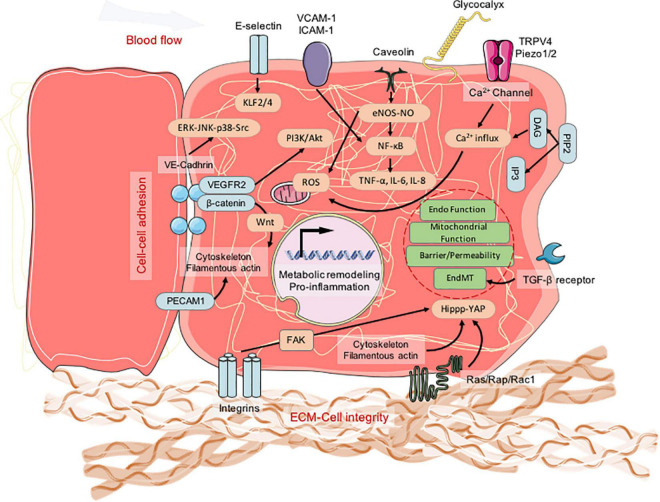
The molecular signaling of mechanotransduction in pulmonary blood vessel endothelial cells. ECM, extracellular matrix.

The mechanical forces associated with cyclical stretching and shear stress play a key role in regulating vascular function and homeostasis of pulmonary circulation. The pulmonary microvascular endothelium is exposed to continuous or periodic stretching during spontaneous breathing and blood flow. Shear stress, stiffness, and cyclic stretch modulate the function and metabolic status of the endothelial cells. The lung microvasculature is subjected to mechanical forces including shear stress and cyclic stretch, which vary with the cardiac and breathing cycles. Endothelial cells are continuously subjected to shear stress that range from 10 to 50 dyne/cm^2^ in the large arteries, 5 to 20 dyne/cm^2^ in the microvasculature, and 10-fold lower in the veins compared to the arteries (Paszkowiak and Dardik, 2003). Physiological circulatory stretching increases the expression of the tight junction-associated protein, occludin, which strengthens the endothelial barrier and upregulates the expression of integrins in the endothelial cells. This further enhances cell adhesion in the EC monolayer and increases the resistance to hemodynamic forces or excessive vasodilation (Figure 3).

#### Ca^2+^ Signaling

In the pulmonary vascular endothelial cells, Ca^2+^ permeable non-selective cationic stretch-activated ion channels (SACs) share similar electrophysiological properties such as linear I–V relationship of the evoked currents with a reverse potential about 0 mV and unitary conductance around 30 pS (Ducret et al., 2010). Transient receptor potential (TRP) channels represent a superfamily of non-selective cationic channels that play a significant role in the endothelial cells. TRPV channels such as TRPV1 and TRPV4 are expressed in the PAECs (Barbeau et al., 2021). TRPV1-activated signaling pathways in the PAECs provide counterbalancing effects at the site of the blood vessel. Furthermore, TRPV4 is widely expressed in every layer of the pulmonary artery (PA) and participates in maintaining the normal biological functions of the vessels. In the PAECs, TRPV4 plays a key role in vasodilation *via* nitric oxide (NO) signaling and endothelium-derived hyperpolarizing factor (EDHF) (Sukumaran et al., 2013). Furthermore, TRPV4 interacts with the endothelial nitric oxide synthase (eNOS) in the PAECs and induces the release of NO by activating unitary Ca^2+^ influx that stimulates the guanylyl cyclase–protein kinase G pathway (Ottolini et al., 2020). Furthermore, mitochondrial impairment in PH causes accumulation of reactive oxygen species (ROS), which induce TRPV4 mediated Ca^2+^ influx. TRPC1 is expressed in the PASMCs and PAECs of rats, mice, and humans (Malczyk et al., 2013). TRPC1 is overexpressed in the PH models. In the pulmonary endothelial cells, TRPC4 regulates microvascular permeability, agonist-dependent vasorelaxation, and gene transcription (Firth et al., 2007). TRPP1 and TRPP2 are both expressed in the vascular smooth muscles and ECs of the cerebral and mesenteric arteries, and regulate blood vessel functions and myogenic tone. However, the roles of TRPP channels in the pulmonary vasculature are not known and require further investigations.

In mammals, two Piezo proteins, namely, Piezo1 and Piezo2 have been reported. Deletion of the Piezo1 gene in mice causes aberrant vascular development resulting in early embryonic death around day 10 (Li et al., 2014). Endothelial Piezo1 channels are necessary for flow-induced vasodilatation through eNOS activation and release of NO (Wang et al., 2016). Besides, NO production is also induced by ATP release through the pannexin channels, which activates the P2Y2 receptors and eNOS (Wang et al., 2016). By contrast, in the mesenteric vessels, Piezo1 induces flow-sensitive cationic ion influx in the ECs of the mesenteric vessels and depolarizes the membrane. This depolarization spreads to the adjacent PASMCs and activates the voltage gated Ca^2+^ channels, thereby inducing vasoconstriction (Rode et al., 2017). Kang et al. (2019) reported that genetic deletion or pharmacological inhibition of Piezo1 reduced the endothelial sprouting and lumen formation when induced by shear stress and the proangiogenic mediator, sphingosine 1-phosphate. Yoda1 enhanced sprouting angiogenesis by activating Piezo1. Physical stimuli triggered Piezo1-mediated Ca^2+^ influx and activated matrix metalloproteinase (MMP)-2 and membrane type I MMP, both of which synergistically facilitated sprouting angiogenesis (Kang et al., 2019). Piezo2 is also present in the human PAECs, but its function is currently unknown (Lambert et al., 2018).

#### Krüppel-Like Factor 2 and Krüppel-Like Factor 4

The Krüppel-like factor (KLF) family of transcription factors regulate integral EC functions, including growth, inflammation, migration, proliferation, cell differentiation, plasticity, and apoptosis (Chang et al., 2017). KLF2 and KLF4 are master transcription factors that regulate vasodilatory, anti-inflammatory, and antithrombotic properties of the quiescent endothelial cells (Denis et al., 2019). KLF2 and KLF4 are upregulated by disturbed flow, and subsequently suppressed PFKFB3 and PFK1, two critical proteins involved in glycolysis (Doddaballapur et al., 2015). Decreased expression of KLF2 or KLF4 induces the production of inflammatory cytokines in the endothelial cells. Stable blood flow reduces glycolysis in a KLF2-dependent manner, and increases mitochondrial biogenesis through PPARγ/PGC1 signaling (Pollak et al., 2018). Furthermore, unidirectional flow promotes the degradation of HIF-1α, which inhibites pyruvate dehydrogenase kinase 1 (PDK1) and increases mitochondrial complex I activity. Slegtenhorst et al. (2018) reported the endothelial atheroprotection role of KLF2 and its inducer, Simvastatin.

#### Integrins-Cytoskeleton-Lamin A

Cells respond to external forces through integrin-mediated remodeling of the ECM (Sun et al., 2016). Stretch-induced activation promotes interactions between integrins and focal adhesion proteins, thereby converting the mechanical signal into biochemical cascades. In the endothelial cells, integrins α2 and β1 stimulated the p38-MAPK signaling pathway (Bix et al., 2004). Focal adhesion integrates the actin cytoskeleton with the ECM interface and helps maintain the endothelial cell barrier integrity. Increased focal adhesion triggers the activation of small GTPase and Rho kinase signaling pathways. High magnitude cyclic stretching (18%) stimulates the formation of focal adhesion complexes that included paxillin, ERK1/2, MAPK, NF-κB, RhoA, and GEF-H1. Moreover, focal adhesion is redistributed under shear stress (15 dyn/cm^2^). In vascular endothelial cells, VE-cadherin acts as a link between the cytoskeleton and the adherens junctions (Fang et al., 2019). VE-cadherin regulates the cellular orientation of endothelial cells in response to shear stress through PECAM-1, VEGFR2, and PI3K signaling (Tzima et al., 2005). Endothelial cells also sense and transmit the mechanical force-induced signals *via* gap junction-mediated propagation of Ca^2+^ signaling. Connexin-32 responds to mechanical stimulation by generating intracellular Ca^2+^ waves through *N*-cadherin (Ko et al., 2001). Hence, the interaction between gap and adherens junctions is critical for establishing cell–cell communications. The cytoskeleton plays a critical role in transducing mechanical stress-related signals. Deguchi et al. (2005) demonstrated that the force should be balanced between the basal actomyosin stress fibers and focal adhesion complexes in the endothelial cells. Depolymerization of F-actin, ROCK inhibitor, and PKA activation results in actin disassembly, attenuation of actomyosin assembly, and stress fiber formation. Cytoskeletal changes result in the activation of RhoA and Rac GTPases, which promote cellular reorientation and transcription responses by altering lamin A; knock-down of lamin A abolishes the changes of histone deacetylases (HDAC), thereby demonstrating the role of lamin A in regulating the chromatin state (Nayebosadri and Ji, 2013).

#### Rho GTPase-YAP/TAZ

Endothelial barrier regulation is dependent on the cytoskeletal rearrangements (Vogel and Malik, 2012). The small Rho GTPases, RhoA, and Rac1, are central regulators of vascular permeability through cytoskeletal reorganization (Asano-Matsuda et al., 2021). RhoA and Rac1 exert opposing functional effects. RhoA activation promotes endothelial contraction and induces barrier disruption, whereas Rac1 stabilizes the endothelial junctions and increases barrier integrity. Ke et al. (2019) demonstrated a key role for RhoA GTPase in high cyclic stretch-induced endothelial cell barrier dysfunction. Besides, Rap1 GTPase is also involved in the regulation of cytoskeleton and cell junctions. Rap1-mediated signaling induced lung vascular EC barrier restoration; inhibition of Rap1 activity enhanced ventilator-induced lung injury (VILI) at both low- and high-volume ventilation conditions (Ke et al., 2019). The Hippo/YAP signaling pathway is also involved in mechanotransduction. YAP and TAZ are activated by stiff ECM and serve as a central regulatory hub for cellular proliferation and survival in multiple organs during tissue growth and development (Dupont et al., 2011). Furthermore, ECM stiffening promotes vascular cell growth and migration *via* YAP/TAZ-dependent glutaminolysis and anaplerosis, thereby linking mechanical stimulation to vascular metabolic dysregulation (Bertero et al., 2016). Therefore, YAP/TAZ pathway is a potential metabolic drug target in PH therapy. Greater stiffnesses (around 50 kPa) increases the proliferation and migration of endothelial cells through the YAP/TAZ signaling pathway (Bertero et al., 2016), TGF-β, Toll-like receptors, and NF-κB (Thenappan et al., 2018). NF-κB functions as a nuclear effector that integrates signals from multiple signaling pathways. Membrane receptors such as Toll-like receptors (Davidovich et al., 2013; Liu et al., 2013) stimulate the expression of MCP-1, a potent chemoattractant for monocytes, and increase the expression of IL-6 and COX-2. The activation of NF-κB is mediated by the integrins through the PI3K-PLC-PKC signaling cascade (Mussbacher et al., 2019).

#### Metabolic Status

Mitochondria anchor to the cytoskeleton and function as mechanotransducers by releasing ROS in response to the cytoskeletal strain (Ali et al., 2004). Besides, ROS play a key role in the activation of NF-κB and VCAM-1. In the ECs, mitochondria-derived H_2_O_2_ diffuses into the cytosol in response to shear stress and initiates oxidative signaling that upregulates hemeoxygenase(HO)-1 and maintains the atheroprotective EC status (Han et al., 2009). NADPH oxidases (NOXs), mitochondria, and xanthine oxidases are the main sources of superoxide in response to mechanical stress (Ichimura et al., 2003; McNally et al., 2003). Stretch-induced ROS production in the endothelium upregulates the expression of cell adhesion molecules and chemokines in collaboration with several ROS-generating enzymes such as NADPH oxidases and eNOS. In the pulmonary endothelial cells, NO levels are increased through PI3K, AKT, and eNOS. Moreover, xanthine oxidoreductase (XOR) is activated by the p38 and ERK1/2-MAPK signaling pathways. ROS signaling is regulated by cyclic stretch in an amplitude-dependent manner and plays a critical role in various EC responses to cyclic stretch. Long-term cyclic stretching (5–12%) caused magnitude-dependent downregulation of Nox4, Cu/Zn superoxidase dismutase (SOD), MnSOD, catalase, and ROS (Goettsch et al., 2009). Cyclic stretching regulates survival and angiogenesis of endothelial cells *via* NOX-induced ROS.

Yamamoto et al. (2020) used real-time imaging technology to demonstrate the novel role of endothelial mitochondria in transducing shear stress signals by triggering ATP generation and release, and purinoceptor-mediated Ca^2+^ signaling. Glycolysis is a crucial metabolic pathway that converts glucose first to pyruvate and then to lactate. Endothelial cells are highly glycolytic (Wu and Birukov, 2019). Furthermore, the glycolytic index (lactate/glucose ratio) in cultured human umbilical vein endothelial cells (HUVECs) is around 1.74, which suggests that the endothelial cells metabolize glucose entirely into lactate (Kim et al., 2017). Thus, only a small fraction of the glycolytic intermediate pyruvate is metabolized by the mitochondrial OXPHOS. Endothelial cells, in contrary to other cell types, are as glycolytic as the tumor cells (De Bock et al., 2013a), and use very little oxygen to generate ATP (De Bock et al., 2013b). Therefore, glycolysis plays a critical role in nucleic acid synthesis and survival, whereas electron transport chain-dependent mitochondrial metabolism is required for cell proliferation, angiogenesis, and redox balance.

## Pathological Mechanical Stress Induces Pulmonary Diseases

### Ventilation Induced Lung Injuries and Acute Respiratory Distress Syndrome

Exposure to non-physiological stretch disrupts the normal mechanical stress response mechanisms and triggers aberrant repair mechanisms during lung injury. Mechanical ventilation induces or exacerbates ventilation-induced lung injury (VILI). Dagenais et al. (2018) showed that scratching the AEC monolayer generates a TRPV4-dependent Ca^2+^ wave, which spreds from the margin of the scratch to the distant cells and induces abnormal activity of the epithelial sodium channel, ENaC; moreover, TRPV4 agonist, GSK1016790A, abolishes the Ca^2+^ wave and other downstream signaling events (Dagenais et al., 2018). Diem et al. (2020) demonstrated that small plasma membrane invaginations called caveolae in the AT1 cells play a key mechanotransductive role in the AT1 cells by responding to mechanical stress at the plasma membrane; these caveolae are absent in AT2 cells. Ventilation-induced stretch stimulates Ca^2+^ entry *via* caveolae-resident Piezo1-activated pannexin-1 hemichannels and results in ATP release from the AT1 cells. The released ATP triggers Ca^2+^ influx in the neighboring AT2 cells and induces inflammation by recruiting the monocytes. Liang et al. (2019) reported increased expression of Piezo1 in the AT2 cells mediates Ca^2+^ influx and triggers Bcl-2 dependent apoptosis during ARDS, but these effects are abrogated by inhibiting Piezo1. Since the vascular endothelial cells are subjected to cyclic stretch during alveolar movement, they are prone to damage during VILI. Zhang et al. (2021) reported higher Piezo1 expression in the pulmonary endothelial cells in response to high tidal volume mechanical ventilation and cyclic stretching. Enhanced expression of RhoA/ROCK1 in the endothelial cells subjected to cyclic stretch or Yoda1 treatment is significantly abrogated by Piezo1 deficiency or inhibition of Piezo1. Furthermore, inhibition of RhoA/ROCK1 signaling does not affect Piezo1 expression, but the inhibition of Piezo1 by GSMTx4 alleviates VILI-induced pathological changes (Zhang et al., 2021).

Inflammation is one of the major causes of lung injury. High tidal volume mechanical ventilation induces significant changes in microvascular permeability, neutrophil infiltration, levels of malondialdehyde, macrophage inflammatory protein-2 (MIP)-2, and NF-κB, and the activation status of the NF-κB repressing factor (NKRF). Yehya et al. (2019) showed that the human epidermal growth factor receptor 3 (HER3) ligand neuregulin-1 (NRG1) enhances VILI by activating HER2 and induces increased permeability and upregulation of claudin-7. HER2 activates the IL-6 receptor and the IL-6 inflammatory loop, which contributes to lung injuries. Ning et al. (2012) shows that mechanical stretch induces early apoptosis and IL-8 secretion in the AT2 cells. Furthermore, mechanical stretch upregulates ER stress and increases the expression levels of monocyte chemoattractant protein (MCP)-1/C-C motif chemokine ligand 2 (CCL2) and MIP-1β/CCL4 pro-inflammatory chemokines in the AT2 cells, thereby enabling monocyte recruitment (Valentine et al., 2018). Furthermore, EC-derived microparticles (EMPs) are released during significant lung inflammation and injury. Zhang et al. (2014) demonstrated that during mechanical ventilation-induced VILI, NLRP3 inflammasomes released by the endothelial or epithelial cells mediated the recruitment of pulmonary macrophages and induced autophagy in the lung epithelial cells. In the *in vivo* experiments, mechanical ventilation induces lung leukocyte recruitment as well as accumulation of cells and cytokines in the alveolar space. Cyclic stretch-induced endothelial cells activated gVPLA2, which enhances the expression levels of intercellular adhesion molecule 1 (ICAM-1) and promotes the adhesion of polymorphonuclear neutrophils to the EC, thereby inducing EC injury (Meliton et al., 2013).

Ding et al. (2012) showed that mechanical stretch significantly enhances MAP2K6 activity and HMGB1 protein expression in alveolar epithelial cells. Wolfson et al. (2014) reported increased HMGB1 expression in the human lung micro-vessel endothelial cells exposed to excessive mechanical stress *via* STAT3 and Rho GTPase signaling. Gao et al. (2014) demonstrated that pathological cyclic stretching significantly increases lung cell apoptosis by repressing Rac and increasing Rho expression levels.

Pathological elevation of lung vascular pressure or regional or generalized overdistension of pulmonary microvascular and capillary beds caused by mechanical ventilation at high tidal volumes are two commonly encountered clinical scenarios in lung diseases. Elevated mechanical strain on the lungs increases the production of ROS in both endothelial and alveolar epithelial cells and causes VILI (Poljsak et al., 2013). Cyclic stretching in AT1 cells increases the ROS levels, which enhances monolayer permeability *via* activation of NF-κB and ERK (Davidovich et al., 2013). These data suggest that antioxidants may prevent or alleviate VILI. Song et al. (2016) shows that rat AT2 cell monolayers generates increased levels of ROS, including NO and superoxide under mechanical ventilation stress. Tanaka et al. (2017) demonstrated that non-physiological cyclic stretch increased oxidative stress by up regulating NOX and DUOX2. Mitochondria-targeted antioxidant MitoTempo significantly reduces oxidative stress and prevents the dissociation of Claudin-4 and Claudin-7 from ZO-1, thereby alleviating VILI (Song et al., 2016). Ge et al. (2019) demonstrates that hydrogen sulfide (H2S) significantly alleviates VILI by inhibiting inflammation and oxidative stress through PERK/eIF2α/ATF4/GADD34 and NF-κB/MAPK pathways. ROS accumulation triggers FasL/Fas extrinsic death pathway in the AT2 cells of newborn rats under prolonged mechanical ventilation (Kroon et al., 2013). miR-135a protects the endothelial cells from pathological mechanical stretching by binding to PHLPP2 and activating the PI3K/AKT pathway (Yan et al., 2018).

### Pulmonary Fibrosis

Pulmonary fibrosis is caused by abnormal tissue repair process driven by the alveolar epithelium, including aberrant fibroblast and myofibroblast proliferation and excessive deposition of ECM. AT2 cells play a key role in regeneration and repair after lung injury because they can differentiate into AT1 cells, the main epithelial cell type at the alveolar–capillary barrier for gas exchange. Hence, impaired renewal capacity of AT2 cells promotes fibrogenesis and the production of profibrotic factors (Selman and Pardo, 2020).

Yes-associated protein 1 plays a key regulatory role in the mechanical tension-induced alveolar regeneration in response to lung injury by activating the CDC42/F-actin/MAPK/YAP signaling cascade (Liu et al., 2016). Activation of YAP suppresses inflammation through IκBα-NF-κB signaling and accelerates alveolar epithelial regeneration and regression of fibrotic lesions. Furthermore, MAPK-mediated activation of YAP promotes alveolar regeneration in response to the mechanical tone of the lung (Liu et al., 2016). YAP also contributes to pulmonary fibrosis by promoting abnormal cell proliferation, migration, and polarity of epithelial cells *via* mTOR/PI3K/AKT signaling (LaCanna et al., 2019). The profibrotic effects of YAP are exerted through its interaction with nuclear transcriptional factors and the activation of genes involved in ECM regulation, such as PAI-1, connective tissue growth factor (CTGF), TGF-β1, COL1A1 and COL1A2 in idiopathic pulmonary fibrosis epithelial cells; YAP also promotes fibroblast growth on stiffness matrix (Giménez et al., 2017; Lee et al., 2020). In pulmonary fibrosis, MRTF-A interacts with the serum response factor (SRF) in the nuclear matrix and promotes transcription of COL1A2 and TGF-β1, which increases the stiffness of ECM (Luchsinger et al., 2011).

Mechanical stretch activates TGF-β1 pathway in the AT2 cells, which alters the homeostatic pulmonary microenvironment leading to aberrant wound healing and tissue fibrosis (Kuhn et al., 2019). In an *ex vivo* model, mechanical tissue stretching induces the activation of TGF-β1 signaling *via* the Rho/ROCK signaling pathway and interactions with αv integrins (Froese et al., 2016). Wu et al. (2020) suggested that increased mechanical tension dysregulates the functions of the AT2 cells and decreases alveolar renewal capacity; tissue stretching during spontaneous breathing results in aberrant activation of the TGF-β1 signaling loop and fibrosis progression (Wu et al., 2020). Furthermore, loss of CDC42 in the AT2 cells promotes periphery-to-center progressive lung fibrosis (Wu et al., 2020). CDC42 maintains the proliferative potential of AT2 cells. Non-physiological mechanical tension activates the TGF-β1 signaling loop in the AT2 cells that drives periphery-to-center progressive lung fibrosis. Besides, *Tgfb1* shRNA treatment significantly reduces the expression of *Tgfb1* in the AT2 cells and the expression levels of type I collagen in the stromal cells. This demonstrates the key function of TGF-β1 in the AT2 cells and fibrosis progression. Moreover, the production of free TGF-β ligands is significantly reduced in the *Cdc42*-null AT2 cells (Wu et al., 2020).

Clinically, ARDS patients who receive mechanical ventilation are prone to lung fibrosis *via* EMT through the Midkine-Notch2-ACE signaling pathway (Zhang et al., 2015). Furthermore, miR-19b overexpression promoted EMT in response to mechanical stretch by down-regulating PTEN (Mao et al., 2017). These studies suggested that EMT played a significant role in lung fibrosis due to mechanical stress. Non-physiological mechanical stretch stimulated excessive ATP release from the lung alveolar cells; ATP induced the release of IL-1β *via* activation of the NLRP3 inflammasome through P2 × 7R receptor binding and facilitated the progression of lung fibrosis (Gicquel et al., 2017). Increased pulmonary vascular pressure induced ROS accumulation due to mitochondrial dysfunction. Moreover, reduced caveolin-1 (CAV1) prevents pulmonary endothelial ROS production with cessation of flow (Milovanova et al., 2008). Mechanical stretching inhibits ERK signaling pathway by inducing the trafficking of CAV1 from the cell membrane to the cytoplasm.

EMT is a mechanism for epithelial remodeling and repair, wherein epithelial cells lose their epithelial characteristics and acquire mesenchymal properties (Rout-Pitt et al., 2018). Therefore, dysregulated EMT promotes pulmonary fibrosis (Hewlett et al., 2018). Mao et al. (2017) shows that miR-19b participates in the EMT process in response to mechanical stretch by activating AKT through inhibition of PTEN. Restoration of PTEN expression or inhibition of AKT phosphorylation suppresses mechanical stretch induced EMT phenotype. Impaired lung mechanics after mechanical ventilation is associated with increased hydroxyproline content of the lung tissues, and increased expression levels of TGF-β, β-catenin, and mesenchymal markers, α-SMA and Vimentin.

Vascular endothelial cells also contribute to pulmonary fibrosis *via* inflammation, metabolic alterations, and endothelial to mesenchymal transition (EndMT). EndMT is dependent on mechanical forces such as shear stress and stiffness. TGF-β-induced EndMT occurs preferentially on stiffer substrates and is inhibited by blocking the β-catenin/Wnt signaling pathway (Zhong et al., 2018). TGF-β-induced EndMT is accompanied by inhibition of fatty acid oxidation, which is required for *de novo* nucleotide synthesis and endothelial cell proliferation (Schoors et al., 2015). Inhibition of fatty acid oxidation reduces intracellular levels of acetyl-CoA, which is required for maintaining the endothelial phenotype of EV cells (Xiong et al., 2018). NLRP3 inflammasome activation contributes to mechanical stretch induced EndMT and pulmonary fibrosis (Lv et al., 2018). Increasing stiffness of lung parenchyma promotes the expression of PF-related factors such as TGF-β and HIF-1α in the endothelial cells (Phan et al., 2021).

### Pulmonary Hypertension

Shear stress, stiffness, and cyclic stretch influence the functional and metabolic states of the endothelial cells. The lung microvasculature is subjected to mechanical forces due to the cardiac output including shear stress and cyclic stretch, which vary according to the cardiac and breathing cycle. Endothelial cells are continuously subjected to shear stress that can range from 10 to 50 dyne/cm^2^ in the large arteries, 5 to 20 dyne/cm^2^ in the microvasculature, and 10-fold lower in the veins compared to the arteries (Paszkowiak and Dardik, 2003). The calculated pressure on the PAECs in the PAH patients is 20.5 ± 4.0 dyne/cm^2^ compared to 4.3 ± 2.8 dyne/cm^2^ in the healthy individuals. The mechanical stress on the endothelial cells persists during disease progression. Clinically, pulmonary arterial hypertension (PAH) involves elevated mean pulmonary arterial pressure, pulmonary artery wedge pressure, and pulmonary vascular resistance (Kovacs et al., 2018). The prevalence of PAH among the pulmonary fibrosis patients is dependent on the severity of pulmonary fibrosis. In the early stages or when initially diagnosed, PAH affects < 10% of patients, but as the disease progresses, the incidence of PAH increases to 32% (Lettieri et al., 2006). Thus, PAH promotes the progression of lung fibrosis by exposing the capillary endothelial cells to higher mechanical stress.

Lhomme et al. (2019) demonstrated that endothelial Piezo1 promotes intrapulmonary vascular relaxation by regulating endothelial Ca^2+^ mobilization and NO production. The inhibition of Piezo1 attenuates the increased expression of NO and Ca^2+^ mobilization. Iring et al. (2019) showed that the endothelial mechanosensitive cation channel Piezo1 mediates fluid shear stress-induced release of adrenomedullin and subsequent Gs-coupled receptor-mediated formation of cAMP that induced eNOS synthase *via* PKA activation; deletion of Piezo1 or adrenomedullin impaires vasodilation and induces hypertension (Iring et al., 2019). Kang et al. (2016) demonstrated that elevated levels of endothelin-1 (ET-1) and increased proliferation of PAECs in the PAH patients is regulated by PPARγ. YAP/TAZ signaling pathway is involved in the responses of endothelial cells to mechanical stress. For example, *in vitro* experiments showed that cells exposed to higher stiffness (50 kPa) increased glycolysis *via* YAP/TAZ/Hippo signaling pathway. Therefore, activation of YAP/TAZ increases the proliferation and migration of endothelial cells, ECM stiffness, and metabolic shift from OXPHOS to glycolysis (Wang and Valdez-Jasso, 2021; Woodcock et al., 2021). NOTCH1 is downstream of bone morphogenetic protein receptor type 2 (BMPR2), which is implicated in PAH through enhanced glycolysis and histone acetylation; BMPR2 induces mitochondrial dysfunction in the endothelial cells and is required for NOTCH1 activation (Liu et al., 2017).

Furthermore, several miRNAs regulate the apoptosis of endothelial cells and play a role in PF. MiR-371b-5p increases proliferation of pulmonary artery endothelial cells (PAECs) *via* PTEN-PI3K-AKT signaling pathway (Zhu et al., 2018). MiR-7 regulates serine and arginine-rich splicing factor 1 (SRSF1), which promotes PAEC migration and increases the stiffness of ECM (Wang and Valdez-Jasso, 2021).

## The Interplay Between Alveolar Epithelial Cells and Vascular Endothelial Cells

### Hypoxia Induced Endothelial Dysfunction

In the lungs, oxygen diffuses from the alveoli into blood circulation and carbon-di-oxide diffuses from the blood into the alveoli. However, gas exchange is impaired when alveolar epithelial cells are injured during pathological conditions such as VILI, ARDS and pulmonary fibrosis resulting in an hypoxic microenvironment. Furthermore, hypoxia causes injury to the vascular endothelial cells and activates HIF-1α expression. HIF-1α regulates critical vascular functions such as angiogenesis, metabolism, cell growth, metastasis, and apoptosis (Semenza, 2017). Increased HIF-1α stabilization reprograms endothelial metabolism and activates vascular inflammation by promoting glycolysis and reducing the mitochondrial respiratory capacity, thereby increasing NOX4-derived ROS levels and activating the deubiquitinating enzyme, Cezanne (Wu et al., 2017).

Emerging evidence suggests that biomechanical stimuli also regulate HIF-1α. In the vascular endothelium, disturbed blood flow significantly stabilizes HIF-1α even under normoxic conditions. YAP/TAZ pathway is also involved in the metabolic homeostasis mechanisms of the PAECs. YAP and HIF-1α promote glycolysis co-operatively because YAP localizes to the nucleus and prevents HIF-1α degradation (Zhang et al., 2018). In ARDS, insufficient oxygen levels promote HIF-1α-dependent elevation of lactate levels due to increased glycolysis; moreover, HIF-1α participates in the upregulation of TNF-α, IL-6, and IL-8. Pathological mechanical stress also activates HIF-1α, which protects the endothelial barrier by regulating VEGFR2 and the vascular endothelial protein tyrosine phosphatase (VE-PTP) that dephosphorylates TIE2 and ANG2. Several lncRNAs (n335470, n406639, n333984, and n337322) also regulate pulmonary inflammation and fibrosis induced by cyclic stretch through hypoxia and NF-κB signaling (Wang D. et al., 2021).

Long-term exposure to a hypoxic environment alters the redox balance and increases cellular inflammation *via* increased expression of MIP2, IL6, TNF-α and CXCL1, as well as elevated oxidative stress and apoptosis. Furthermore, ICAM1, vascular cell adhesion molecule 1 (VCAM1), and selectin mediate the interactions between the monocytes and endothelial cells *via* the NF-κB-ERK signaling pathway (Wohlrab et al., 2018).

### Endothelial Permeability Impairment Causes Fluid Leaking

Endothelial cell injury plays a significant role in the pathology of VILI and ARDS. Pulmonary edema is caused by impaired cytoskeleton and permeability of the endothelial cells. Zeinali et al. (2021) developed an *in vitro* three-dimensional (3D) micro-vessel model to investigate the effects of the 3D mechanical cyclic stretch of different magnitudes and vascular endothelial growth factor (VEGF) stimulation on a 3D perfusing vasculature; the results shows that physiological cyclic stretch restored the vascular barrier tightness and significantly decreases vascular permeability (Zeinali et al., 2021). Piezo1 activation and calpain-induced disruption of VE-cadherin adhesion in endothelial cells subjected to elevated lung micro-vessel pressure resulted in capillary stress failure and edema (Friedrich et al., 2019). Dopamine D1 receptor (DRD1) is downregulated in both surgical patients and mice exposed to mechanical ventilation. The administration of DRD1 agonist attenuates the mechanical stretch-induced lung endothelial barrier dysfunction by inhibiting deacetylation of α-Tubulin *via* cAMP/EPAC/HDAC6 signaling pathway (Wang Y. et al., 2021). Vascular homeostasis is regulated by normal shear stress sensing and barrier function, which are controlled by the adherens junctions (Stanicek et al., 2020). Endothelial glycocalyx plays a critical role in maintaining capillary fluidity and perfusion homogeneity in the microvasculature by interacting with focal adhesion proteins (Lohser and Slinger, 2015; Knoepp et al., 2020). This suggests a connection between mechanical sensing, NO production, and microvascular perfusion. Damage to the glycocalyx layer causes endothelial dysfunction because of actin cytoskeleton remodeling. Stanicek et al. (2020) demonstrated that lncRNA-LASSIE is associated with the platelet endothelial cell adhesion molecule-1 (PECAM-1) and the intermediate filament protein, Nestin; lncRNA-LASSIE deletion reduces the interaction between VE-cadherin and Nestin, thereby destabilizing the cytoskeleton (Stanicek et al., 2020). Hence, lncRNA-LASSIE is important for the regulation of barrier function. The pathobiology of VILI and ARDS involves increased lung vascular permeability and alveolar flooding because the endothelial cells lose barrier integrity. MMP-2, MMP-9, RGD-dependent integrins, cell-cell adhesion proteins, ICAM-1, VCAM-1, and VE-cadherin play an integral role in maintaining endothelial barrier function (Wang et al., 2017).

### Extracellular Matrix Stiffness Alters Lung Microenvironment

Substrate stiffness plays an important role in regulating tissue-specific endothelial response to shear stress. The physiological stiffness of lung tissue is around 1kPa. Endothelial cells subjected to higher shear stress exhibit cell quiescence marked by lower expression of inflammatory markers and higher NO levels, whereas, ECs subjected to low shear stress demonstrate activated pro-inflammatory state and low NO levels. Cellular traction stress should match substrate stiffness through force sensing at the focal adhesion; therefore, larger tensile stress is necessary to overcome substrate stiffness (Califano and Reinhart-King, 2010). Hemodynamic shear sensors are activated in response to low mechanical force (Fang et al., 2019). Substrate stiffness promotes EndMT and plays a significant role in chronic lung fibrosis diseases, which originate from AEC injury. Elevated ECM stiffness is an independent predictor of cardiovascular morbidity and mortality (Smulyan et al., 2016). Endothelial cells subjected to shear stress demonstrate decreased expression of αv and β3 integrins, which promote migration and elongation *via* EndMT. ECM stiffness in cultured PAECs increases glycolysis and glutaminolysis while reducing mitochondrial oxygen consumption. Furthermore, stiff ECM promotes proliferation and collagen deposition in the PAECs (Bertero et al., 2016). Stiffness is also linked to metabolic signaling through HIF-1α in the pulmonary microvasculature. Hypoxic metabolic modeling of endothelial cells promotes collagen deposition in a HIF-1α-dependent manner (de Jong et al., 2016). Besides, YAP/TAZ pathway activated by high stiffness promotes fibrotic signaling pathways that increase the synthesis of ECM proteins (Totaro et al., 2018). Besides, increased levels of miR-143-3p in ECs under shear stress induces the release of TGF-β in collaboration with SRF and ECM reorganization by targeting collagen V-α2 biosynthesis (Troidl et al., 2020). Therefore, increased ECM stiffness is observed in both epithelium or endothelium injuries, and alters transcription, metabolism, and inflammation in both alveolar epithelial cells and endothelial cells.

### Metabolic Disorders and Oxidative Stress

Laminar shear stress generated by the blood flow stimulates endothelial cells and activates signal transduction pathways that play a significant role in vascular homeostasis (Hirata et al., 2021). Pathological mechanical stress alters the metabolic status of pulmonary endothelial cells. Functional lipidomics of human PEACs showed that laminar shear stress for 24 h significantly alters the levels of 198/761 (26%) species of lipids (Hirata et al., 2021). Lipid changes in pulmonary endothelial cells stimulated the pro-inflammatory response by inducing the expression of VCAM-1. Besides, fragmented phospholipids generates by phospholipid oxidation and nitroxidative stress induces endothelial barrier dysfunction *via* pro-inflammatory cytokines (Hirata et al., 2021). Shear stress also decreases cholesterol in the plasma membrane, but these effects are secondary to the release of ATP. *In vitro* experiments demonstrates that addition of cholesterol to pulmonary cells restores mitochondrial function including ATP production. Furthermore, excessive release of ATP by the alveolar epithelial cells or the endothelial cells affects the lung micro-environment and influences the functions of both pulmonary cell types. Moreover, changes in the levels of metabolic compounds such as cholesterol within the lung micro-environment impaired the normal communication between the epithelium and endothelium (Yamamoto et al., 2020).

Metabolic hemostasis is altered during pathological shear stress. The abnormal blood flow reduces mitochondrial mass and function and upregulates glycolysis through HIF-1α activation; this results in increased accumulation of ROS and defective synthesis of NO. Elevated levels of ROS prevent the degradation of HIF-1α through a positive feedback mechanism and promote the activation of glycolytic genes (Kim et al., 2017; Wu et al., 2017). In mechanically ventilated septic patients, nitroxidative stress increases NO production, protein nitration, and lipid peroxidation. Besides, unidirectional flow increases oxidative phosphorylation (OXPHOS) and mitochondrial biogenesis *via* SIRT1, a key regulator of NOS activity (Wu and Birukov, 2019). Mitochondrial dysfunction increases localized oxidative stress and stimulated hypoxia. Therefore, injury to either alveolar epithelial cells or PAECs increases ROS levels and hypoxia in the microenvironment, which negatively impacts both cell types. Arachidonic acid (AA) significantly increases cellular stiffness. AA metabolites such as prostacyclins and epoxyeicosatrienoic acids are involved in vascular dilation; AA is metabolized to prostacyclin and epoxyeicosatrienoic acids by COX and cytochrome P450 epoxygenases, respectively (Merna et al., 2018). Increased vascular oxidative stress induces non-enzymatic production of isoprostanes from AA. The vasoconstrictor metabolites of AA and isoprostanes induced endothelial damage and impair vascular function. Therefore, oxidative stress alters the balance between vasodilator and vasoconstrictor metabolites of AA.

Shear stress modulates mitochondrial ATP production in vascular endothelial cells by triggering ATP release and Ca^2+^ signaling *via* purinoceptors (Yamamoto et al., 2020). However, abnormal Ca^2+^ signaling induces mitochondrial dysfunction. Lu et al. (2021) demonstrated that inhibition of TRPV4 disrupts the PAEC barrier *via* PKC dependent phosphorylation of Threonine 495 in eNOS. Uncoupling of eNOS promotes mitochondrial redistribution and impairs mitochondrial bioenergetics. Furthermore, acetylation is critical for the stability of the endothelial cytoskeleton. Acetylation of α-tubulin promotes microtubule stability (Kull and Sloboda, 2014; Szyk et al., 2014). Fatty acid-derived acetyl-CoA is a major regulator of cellular acetylation. Impaired mitochondrial function suppresses the acetylation levels in the pulmonary cells. The levels of HDAC6, which is involved in acetylation *via* the canonical Wnt/β-catenin pathway, are elevated in lung injury caused by disassembling the adherens junctions.

### Activation of Inflammation and Monocyte/Macrophage Recruitment

Mechanical ventilation promotes acute lung injury and development of multiple organ dysfunction syndrome by increasing the levels of TNF-α, IL-1β, IL-6, IL-10, MIP-2, and interferon-γ in the lavage fluid (Belperio et al., 2006). Stretching induces the production of TNFα, IL-8, and IL-6 by lung- resident macrophages and AT2 cells; whereas, exposure of lung endothelium to 20% cyclic stretch upregulates the levels of IL-8, VCAM-1, ICAM-1, and *E*-selectin, and mediates the adhesion of monocytes and macrophages (Iwaki et al., 2009).

Accumulation of monocytes and macrophages in the perivascular and adventitia space is a notable feature of remodeling in response to lung injuries (Stenmark et al., 2013). Monocytes and macrophages play a central role in local lung inflammation as a result of PH (Chen et al., 2016), and are associated with disease severity and progression (Willis et al., 2018). Dysregulation of chemokines such as CCL5, CCL2, and CXC3CL1, and their homologous receptors are related to the pathogenesis of PH because the infiltration of monocytes, macrophage polarization, and vascular remodeling in the lungs is regulated by these chemokines (Groth et al., 2014). Hypoxia-induced PH increases the expression levels of CX3CR1, CCR2, and their corresponding ligands, CX3CL1 and CCL2, in the mouse pulmonary vessels; moreover, CX3CR1 deficiency increases the proportion of monocytes and macrophages in the lungs and promotes M2 to M1 macrophage polarization, a classic activating proinflammatory phenotype (Amsellem et al., 2017). Increased strain and frequency of cyclic stretch promotes the secretion of pro-inflammatory factors by the lung-resident macrophages. For example, 12% stretching or elongation of the membrane increases the production of proinflammatory cytokines, such as TNF-α, IL-6, IL-8, and MMP9 *via* NF-κB activation by the human alveolar macrophages. Furthermore, murine alveolar macrophages subjected to 20% cyclic stretch induces the release of IL-1β and IL-18 as well as inflammasome activation through ROS-mediated caspase 1 and TLR4 signaling (Wu et al., 2013).

The dysfunction of alveolar epithelium induced inflammation responses and disturbed the blood flow leading to the downregulation of KLF2 *via* glycocalyx sensing mechanotransduction (Huang et al., 2017). Growth differentiation factor-15 (GDF-15), also known as macrophage inhibitory cytokine-1 or non-steroidal anti-inflammatory drug-activated gene has been identified as a biomarker of treatment response and prognosis in cardiovascular diseases. GDF-15 is a member of the transforming growth factor-β superfamily and participates in several pathological conditions such as inflammation, cancer, as well as cardiovascular, pulmonary, and renal diseases (Arkoumani et al., 2020). Endothelial cells are the source of GDF-15, which interacts with the proinflammatory cytokines and induces localized macrophage accumulation and fibrosis. The activation of host monocytes and macrophages *via* NF-κB signaling induces fibrosis and alveolar epithelium injuries through elevated stiffness of the ECM. Stiffness also increases endothelial inflammation *via* NF-κB through a positive feedback mechanism, thereby enhancing lung fibrosis.

## Future Aspects and Conclusion

In summary, mechanical stress and its related transduction pathways demonstrates a critical molecular biological function in development, functional maturation and pathogenesis. Typically, lung serves as the places for gas exchange, alveoli and blood vessels are essential for such biological process. The epithelial cells and endothelial cells would both undergo mesenchymal transition under various injuries. Besides, the two types of cell are all sensitive to mechanical stress. Cyclic stretch is the major source of mechanical stimulation on alveolar epithelial cells, while cyclic stretch and shear stress are loaded on vascular endothelial cells in general. In addition, the stiffness of the epithelium and endothelium is also essential to maintain the microenvironment for gas exchange. In this review, we summarized the kinds of mechanical stresses that are applied to alveolar epithelial cells and endothelial cells. We demonstrated pulmonary inflammation activation, metabolic alternation, ECM and cytoskeleton remodeling during non-physiological mechanical stress. However, the communications between the microenvironment, the alveolar epithelial cells and the vascular endothelial cells are rarely analyzed. Therefore, we propose a mechanistic picture in which several links are orchestrated for the interplays between epithelium and endothelium, and to emphasize the possibility of targeting the communications in dealing with lung injuries. In the future, more efforts should be directed toward further elucidation of the regulatory mechanisms of these communications, and corresponding research and discovery of novel targets on mechanotransduction signaling as medication therapeutics. With the newly invented methods to deliver nucleotides into lung tissues, it is believed that eventually mechanical pathways under pathological circumstances may be rectified by genetic editing and gene therapy, which seems to be a promising strategy for exploring new cures for lung injury and disease.

## Author Contributions

JW and YL conceived of the presented idea. CL, XZ, and SL summarized the reference and drafted the manuscript. YZ organized the figures. YZ, JW, and YL supervised the project and contributed equally to the final version of the manuscript. All authors contributed to the article and approved the submitted version.

## Conflict of Interest

The authors declare that the research was conducted in the absence of any commercial or financial relationships that could be construed as a potential conflict of interest.

## Publisher’s Note

All claims expressed in this article are solely those of the authors and do not necessarily represent those of their affiliated organizations, or those of the publisher, the editors and the reviewers. Any product that may be evaluated in this article, or claim that may be made by its manufacturer, is not guaranteed or endorsed by the publisher.
